# Pre-operative MRI Radiomics for the Prediction of Progression and Recurrence in Meningiomas

**DOI:** 10.3389/fneur.2021.636235

**Published:** 2021-05-14

**Authors:** Ching-Chung Ko, Yang Zhang, Jeon-Hor Chen, Kai-Ting Chang, Tai-Yuan Chen, Sher-Wei Lim, Te-Chang Wu, Min-Ying Su

**Affiliations:** ^1^Department of Medical Imaging, Chi-Mei Medical Center, Tainan, Taiwan; ^2^Department of Health and Nutrition, Chia Nan University of Pharmacy and Science, Tainan, Taiwan; ^3^Department of Radiological Sciences, University of California, Irvine, Irvine, CA, United States; ^4^Department of Radiology, E-DA Hospital, I-Shou University, Kaohsiung, Taiwan; ^5^Graduate Institute of Medical Sciences, Chang Jung Christian University, Tainan, Taiwan; ^6^Department of Neurosurgery, Chi-Mei Medical Center, Chiali, Tainan, Taiwan; ^7^Department of Nursing, Min-Hwei College of Health Care Management, Tainan, Taiwan; ^8^Department of Biomedical Imaging and Radiological Sciences, National Yang-Ming University, Taipei, Taiwan

**Keywords:** magnetic resonance imaging, radiomics, support vector machine, meningioma, progression, recurrence

## Abstract

**Objectives:** A subset of meningiomas may show progression/recurrence (P/R) after surgical resection. This study applied pre-operative MR radiomics based on support vector machine (SVM) to predict P/R in meningiomas.

**Methods:** From January 2007 to January 2018, 128 patients with pathologically confirmed WHO grade I meningiomas were included. Only patients who had undergone pre-operative MRIs and post-operative follow-up MRIs for more than 1 year were studied. Pre-operative T2WI and contrast-enhanced T1WI were analyzed. On each set of images, 32 first-order features and 75 textural features were extracted. The SVM classifier was utilized to evaluate the significance of extracted features, and the most significant four features were selected to calculate SVM score for each patient.

**Results:** Gross total resection (Simpson grades I–III) was performed in 93 (93/128, 72.7%) patients, and 19 (19/128, 14.8%) patients had P/R after surgery. Subtotal tumor resection, bone invasion, low apparent diffusion coefficient (ADC) value, and high SVM score were more frequently encountered in the P/R group (*p* < 0.05). In multivariate Cox hazards analysis, bone invasion, ADC value, and SVM score were high-risk factors for P/R (*p* < 0.05) with hazard ratios of 7.31, 4.67, and 8.13, respectively. Using the SVM score, an AUC of 0.80 with optimal cutoff value of 0.224 was obtained for predicting P/R. Patients with higher SVM scores were associated with shorter progression-free survival (*p* = 0.003).

**Conclusions:** Our preliminary results showed that pre-operative MR radiomic features may have the potential to offer valuable information in treatment planning for meningiomas.

## Introduction

Meningiomas are the most frequently diagnosed primary brain tumors (1). Although most meningiomas are classified as grade I benign tumors according to the 2016 WHO classification system (2), a subset of these tumors may show early progression/recurrence (P/R) after surgical resection (3–5). Furthermore, the rate of P/R is especially high in cases in which Simpson grade I resection is difficult to achieve, such as for parasagittal and skull base meningiomas (6). Conventional MR imaging findings such as tumor size, bone invasion, and parasagittal location have all been identified as important imaging parameters related to P/R in meningiomas (5, 7). However, most data are presented in qualitative and subjective terms, and interreader inconsistencies may occur during data interpretation.

Radiomics is a new approach in the diagnosis, treatment planning, and prediction of prognosis in brain tumors (8–10). It works by extracting a large number of quantitative characteristics from a medical image and then analyses these features by means of a series of machine learning algorithms (11). Although the radiomics approach for the evaluation of meningiomas pertaining to tumor grades and histological subtypes had recently been reported (12–15), models for predicting clinical outcomes in overall meningiomas are still rare (10, 16). Among the machine learning techniques, several studies had reported that support vector machine (SVM) classifiers offer excellent results in the classification and segmentation in brain tumors (17–22). The purpose of this study is to investigate the role of quantitative radiomics approach based on automatically segmented tumor and SVM classification for the prediction of P/R in meningiomas.

## Materials and Methods

### Ethics Statement

This study was approved by our Institutional Review Board (IRB no.: 10902-009). Written consent was waived because the retrospective nature of this study meant that the healthcare of the included subjects was not affected. Personal information of all included patients was anonymized and de-identified before analyses were carried out.

### Patient Selection

The inclusion criteria were patients diagnosed with WHO grade I meningiomas by means of pathological confirmation. All the included patients must have undergone pre-operative brain MRI, post-operative follow-up brain MRIs for more than 1 year, and at least one MRI performed at 3 to 6 months after surgery. Patients diagnosed with neurofibromatosis (*N* = 3) were excluded. From January 2007 to January 2018, a total of 128 patients (43 men and 85 women with a median age of 57.5 years) diagnosed with WHO grade I meningiomas were included according to the abovementioned criteria. No known history of pre-operative intracranial radiation was documented in any of the included subjects. The mean follow-up time was 64.2 months (ranging from 14 to 149 months). A total of 19 (19/128, 14.8%) patients were found to have P/R, and the mean time to P/R was 33.3 months (ranging from 8 to 92 months). Based on anatomic location, the tumors were classified into four subgroups: convexity, parasagittal and parafalcine (PSPF), skull base, and intraventricular meningiomas. Skull base meningiomas included tumors arising from the anterior fossa/olfactory groove, spheno-orbital region, temporal floor, sellar/cavernous sinus, and posterior fossa (23). The extent of tumor resection was determined by a review of pre-operative brain MRI and the first time post-operative MRI (3–6 months after surgery) by a neuroradiologist (C.C.K.) and a neurosurgeon (S.W.L.). Simpson grade I–III resections (considered gross total resection, GTR) were performed in 93 patients, and Simpson grade IV–V resections (considered subtotal tumor resection, STR) were performed in 35 patients. Post-operative adjuvant radiotherapy (RT) was provided for patients who underwent STR in our institution. A total of 35 patients received post-operative adjuvant RT. Post-operative adjuvant RT was carried out *via* stereotactic radiosurgery (SRS) (*N* = 28, median dose of 25 Gy, ranging from 18 to 30 Gy; median fraction of 5, ranging from 3 to 5 fractions) or fractionated stereotactic intensity-modulated radiotherapy (IMRT) (*N* = 7, dose ranging from 55 to 60 Gy with 30 to 33 fractions) by linear accelerators. Detailed information of post-operative RT protocols is provided in Supplementary File 1 in Supplementary Material.

### Determination of Progression/Recurrence

P/R was evaluated by two experienced neuroradiologists (C.C.K., 7 years of work experience, and T.Y.C., 19 years of work experience) by comparing the post-operative brain MRI findings between the 3–6 months and more than 1 year follow-up. Both readers were blinded to the clinical information of the studied patients. In equivocal cases, final agreement was arrived at by consensus. P/R was defined as recurrence of tumor in Simpson grade I–III resections (GTR) or increasing residual tumor size in Simpson grade IV–V resections (STR) on contrast-enhanced T1WI. In cases of STR, the threshold of P/R was defined as a 10% increase in tumor volume in comparison with post-operative brain MRIs (10). Interobserver reliability in determining P/R with intraclass correlation coefficient (ICC) of 0.8 was obtained. For patients who received post-operative adjuvant RT, P/R was differentiated from post-irradiation effects (pseudoprogression) based on progressive tumor growth, not transient increase in tumor volume (24).

### Imaging Acquisition

Pre-operative brain MRI images were acquired using a 1.5-T (Siemens, MAGNETOM Avanto, *n* = 53, or GE Healthcare, Signa HDxt, *n* = 58) or a 3-T (GE Healthcare, Discovery MR750) (*n* = 17) MR scanner, equipped with eight-channel head coils in each machine. Scanning protocols were as follows: axial and sagittal spin echo T1-weighted imaging (T1WI), fast spin-echo T2-weighted imaging (T2WI), axial fluid attenuated inversion recovery (FLAIR), axial gradient recalled echo (GRE) T2^*^-weighted imaging, axial diffusion-weighted imaging (DWI), and contrast-enhanced (CE) T1WI in axial and coronal sections. Detailed MR imaging parameters can be found in Supplementary File 2 in Supplementary Material.

### Tumor Segmentation

**Figure 1:**
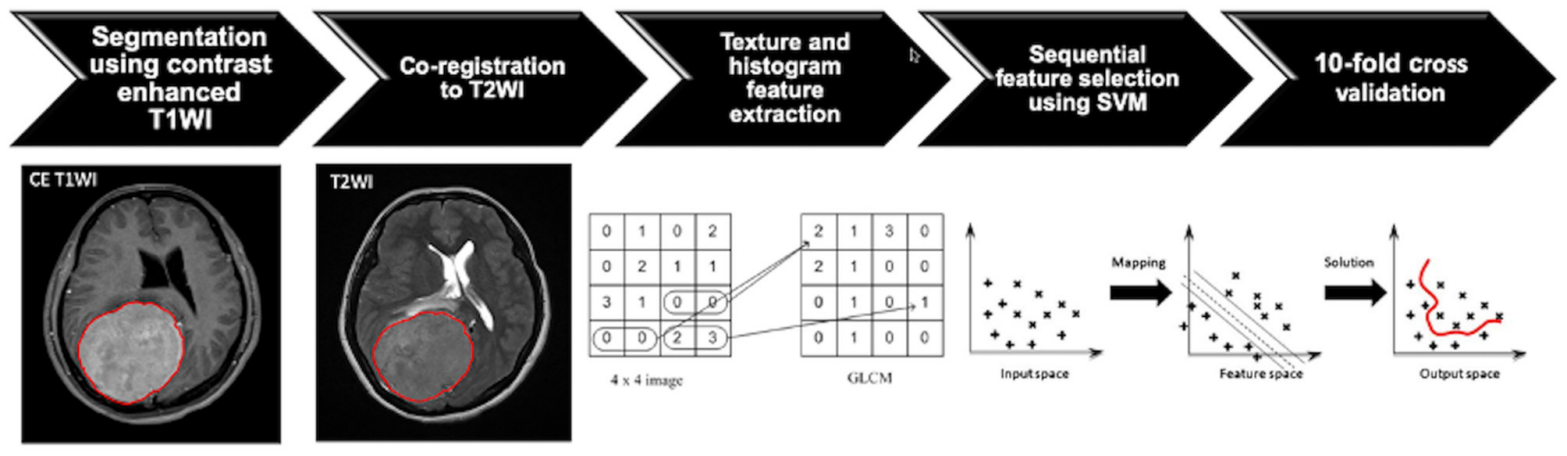
Flowchart indicating the process of analysis for the prediction of progression/recurrence (P/R) in meningiomas. The tumor is first segmented based on contrast-enhanced (CE) T1-weighted image (T1WI), and the region of interest (ROI) of the tumor is then mapped onto the T2-weighted image (T2WI). On each set of the two sequences, a total of 32 first-order features and 75 textural features are extracted, and a total of 214 parameters for each case are collected to develop the classification model. The most important four features are selected by means of the sequential feature selection and support vector machine (SVM) classifiers to calculate SVM score. The 10-fold cross-validation method is applied to test the model performance.

T2WI and CE T1WI were known to be associated with histopathology and tumor grades in meningiomas (8, 25), and the two sequences (slice thickness/spacing, 5 mm/5 mm) were consistently acquired in all subjects. Thus, they were selected for radiomics analysis in this study. Figure 1 shows the flowchart of the analysis process. For each lesion, the operator placed an initial rectangle region of interest (ROI) on axial CE T1WI exhibiting the maximal tumor diameter, locating the approximate location and also deciding the initial and final slices containing the lesion. The fuzzy c-mean (FCM) clustering-based algorithm was developed to calculate the outline of the ROI on each imaging slice (26). In cases of under- or oversegmentation, manual correction by inclusion of more tumor tissue or exclusion of unnecessary normal tissue was performed. After segmentation and correction, the ROIs gleaned from all imaging slices containing the lesion were combined to obtain the 3D information of the whole lesion. The 3D connected component labeling was applied for removing scattered voxels not connecting to the main lesion. The hole-filling algorithm was then applied to include all voxels contained within the main ROI that had been labeled as nonlesion. The final 3D tumor mask was mapped to the axial T2WI to determine the tumor ROI on corresponding imaging slices using affine transformation and linear interpolation by FMRIB's Linear Image Registration Tool (FLIRT) (27).

### Texture Feature Extraction and Selection

Within the segmented tumor on axial CE T1W images and T2W images, 107 imaging features, consisting of 32 first-order features and 75 textural features, were extracted on each modality (Figure 1). Therefore, a total of 214 descriptor features were obtained for each case. In order to evaluate the importance of these features in differentiating P/R, the sequential feature selection process was implemented *via* constructing multiple SVM classifiers (28). Using this method, we selected imaging features with high importance. In this process, SVM with Gaussian kernel was used as the objective function (29, 30). Ten-fold cross-validation was applied to test the model performance (31). In each iteration, the training process was repeated 1,000 times to explore the robustness of each imaging feature. After each iteration, the feature which contributed to the best performance was added into the candidate set. When the addition of features no longer improves the performance, the selection process was terminated and a final set containing the optimal features was obtained. The termination criterion for the objective function was determined at 10^−6^. This procedure was implemented in MATLAB 2018b. The most significant four features selected by the SVM model for the prediction of P/R were T1 gray-level co-occurrence matrix (GLCM) cluster shade, T1 gray-level size zone matrix (GLSZM) gray-level non-uniformity, T2 GLCM cluster prominence, and T2 GLCM cluster shade. The SVM score for each patient was calculated using the following equation based on the selected features.

$$\begin{array}{l} f\left(x\right)=\;\overset{N}{\underset{n=1}{\sum }}w_{n}y_{n}G\left(x_{n},x\right)+b \end{array}$$

where *x* is the input features and *N* is the length of the support vector. *w*_*n*_ is the parameter and *b* is the bias. *y*_*n*_ and *x*_*n*_ are the entries of the supporting vector. *G*(*x*_*n*_, *x*) is the Gaussian kernel function that indicates the dot product in the predictor space between *x* and the support vectors. Herein,

$$\begin{array}{l} G\left(x_{n},x\right)=\;e^{-||x_{n}-x|{|}^{2}} \end{array}$$

### Measurement of Apparent Diffusion Coefficient Value

**Figure 2:**
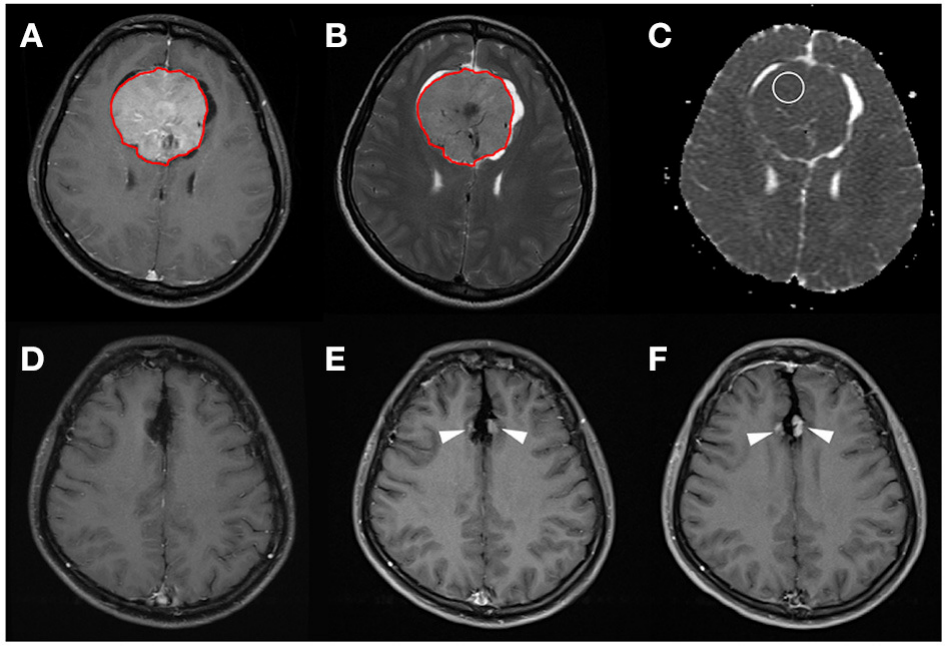
A 31-year-old woman with pathologically proven parafalcine meningioma (WHO grade I). **(A)** Axial CE T1WI showing an enhancing parafalcine tumor (red outline) at the frontal region. The tumor (red outline) is segmented on axial CE T1WI **(A)** and then mapped onto axial T2WI **(B)**. The SVM score based on the four selected radiomic features is 0.831. **(C)** The measured ADC value (circular ROI) is 0.805 × 10^−3^ mm^2^/s (*b* = 1,000 s/mm^2^). **(D)** Gross total tumor resection is performed. **(E,F)** Progressive recurrence of tumor (arrowheads) was observed in 36 months **(E)** and 60 months **(F)** after surgery.

For comparison with the radiomics model in the prediction of P/R in meningiomas, apparent diffusion coefficient (ADC) values (*b* = 1,000 s/mm^2^) on DWI were measured manually by two experienced neuroradiologists (C.C.K. and T.Y.C.) as in previously published works (7, 29). The circular ROI (area ranging from 35 to 78 mm^2^) was placed in a homogeneous area of the tumor to avoid volume averaging with calcification, necrosis, and cystic regions that might influence ADC values (Figure 2) (7, 32, 33). Due to the almost perfect reproducibility in the interobserver reliability, the subsequent statistical evaluation of ADC values was performed using the mean value calculated from both interpreters.

### Statistical Analysis

Statistical analyses were performed using statistical package SPSS (V.24.0, IBM, Chicago, IL, USA). For the evaluation of the clinical parameters and conventional MRI findings, chi-square (or Fisher's exact test) and Mann–Whitney *U* tests were performed for categorical and continuous data, respectively. The area under the receiver operating characteristic curve (ROC) curve (AUC) was calculated for SVM scores and ADC values to obtain the optimal cutoff values. Kaplan–Meier analysis was used to evaluate progression-free survival (PFS), and the log-rank test was used to assess significance. Cox hazard regression model with univariate and multivariate analyses was performed to determine independent predictors of P/R. Variables with a *p* < 0.05 in univariate analysis were brought forward to the multivariate analysis. For multivariate analysis and all other statistical analyses, *p* < 0.05 was considered statistically significant.

## Results

### Clinical Data and Conventional MRI Findings

**Figure 3:**
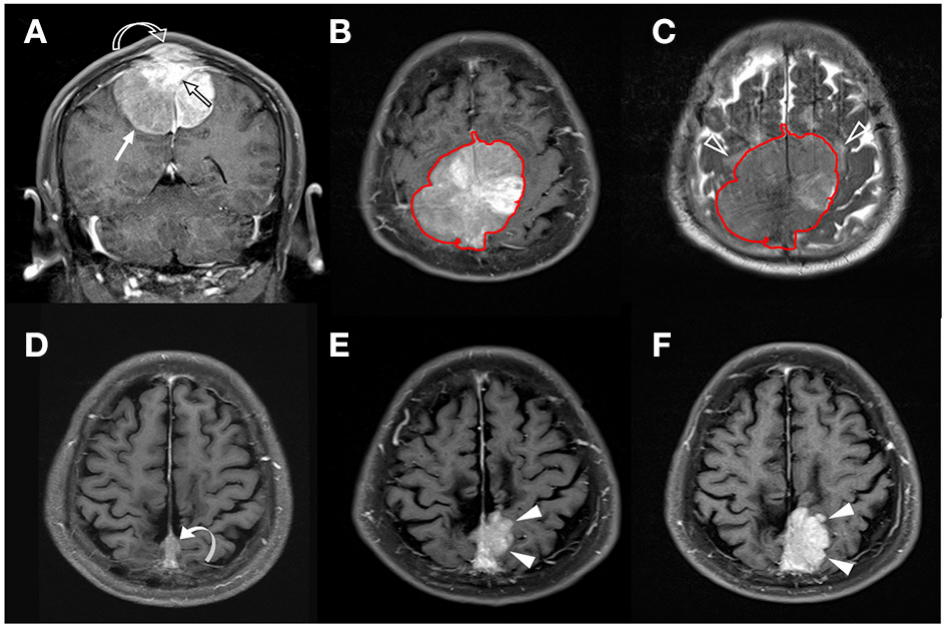
A 58-year-old man with pathologically proven parasagittal meningioma (WHO grade I). **(A)** Coronal CE T1WI shows an enhancing tumor mass (white arrow) in the midline parasagittal region with invasion into the superior sagittal sinus (SSS) (open black arrow) and adjacent skull bone (open curved arrow). **(B,C)** The tumor (red outline) is segmented on the axial CE T1WI **(B)** and then mapped onto the axial T2WI **(C)**. Mild peritumoral edema (white open arrowheads) is noted on T2WI **(C)**. The calculated SVM score based on the four selected radiomic features is 0.337. **(D)** Subtotal tumor resection is performed to preserve the SSS; residual tumor (curved arrow) is noted in the posterior SSS. **(E,F)** Progressive recurrence of tumor (white arrowheads) was observed in 37 months **(E)** and 56 months **(F)** after surgery.

The clinical data and conventional MRI findings of the included 128 meningiomas are summarized in Table 1. Nineteen (19/128, 14.8%) patients were diagnosed with P/R. Statistically significant differences (*p* < 0.05) were observed in the extent of resection, adjacent bone invasion, ADC value, and SVM score between P/R and non-P/R groups (Table 1) (Figures 2, 3). In multivariate Cox hazards analysis (Table 2), adjacent bone invasion, low ADC value, and high SVM score were high-risk factors for P/R (*p* < 0.05) with hazard ratios of 7.31, 4.67, and 8.13.

**Table 1:** Clinical data and conventional MRI findings of meningiomas with and without progression/recurrence (P/R).

	**P/R**	**Non-P/R**	***p-*value**
Number of patients	19	109	
Sex			0.057
Male	10 (52.6%)	33 (30.3%)	
Female	9 (47.4%)	76 (69.7%)	
Age (years)	55 (49.5, 60.5)	59 (52, 66)	0.289
Histological subtypes			0.748
Meningothelial (syncytial)	17 (89.5%)	87 (79.8%)	
Transitional (mixed)	2 (10.5%)	12 (11%)	
Fibroblastic (fibrous)	0	7 (6.4%)	
Angiomatous	0	2 (1.8%)	
Psammomatous	0	1 (0.9%)	
Simpson grade resection			0.007^*^
Grades I, II, and III (gross total resection, GTR)	9 (47.4%)	84 (77.1%)	
Grade IV and V (subtotal resection, STR)	10 (52.6%)	25 (22.9%)	
Post-operative adjuvant RT			0.118
Yes	8 (42.1%)	27 (24.8%)	
No	11 (57.9%)	82 (75.2%)	
Location			0.296
Convexity	4 (21.1%)	30 (27.5%)	
Parasagittal and parafalcine	11 (57.9%)	43 (39.4%)	
Skull base	3 (15.8%)	34 (31.2%)	
Intraventricular	1 (5.3%)	2 (1.8%)	
Peritumoral edema	9 (47.4%)	59 (54.1%)	0.586
Calcification	3 (15.8%)	38 (34.9%)	0.100
Heterogeneous enhancement	7 (36.8%)	46 (42.2%)	0.662
Cystic change or necrosis	3 (15.8%)	19 (17.4%)	1.000
Dural tail sign	11 (57.9%)	65 (59.6%)	0.887
Adjacent bone invasion	8 (42.1%)	7 (6.4%)	< 0.001^*^
Reactive hyperostosis	5 (26.3%)	27 (24.8%)	1.000
Multiplicity	3 (15.8%)	5 (4.6%)	0.096
Maximal diameter (cm)	5.12 (4.22, 6.03)	4.43 (4.09, 4.76)	0.118
Tumor volume (cm^3^)	59.19 (30.35, 88.02)	44.07 (34.96, 53.17)	0.294
ADC value (×10^−3^ mm^2^/s)	0.785 (0.725, 0.845)	0.865 (0.78, 0.95)	0.002^*^
SVM score	0.787 (0.543, 1.032)	0.272 (0.080, 0.464)	< 0.001^*^
Follow-up time (months)	72 (40, 104)	57 (35.2, 78.8)	0.437

*Continuous variables were presented as median and interquartile range (IQR).*

* *Statistical difference (p < 0.05)*.

**Table 2:** Cox proportional hazards analysis for P/R.

	**Univariate analysis**		**Multivariate analysis**	
	**HR (95% CI) for P/R**	***p***	**HR (95% CI) for P/R**	***p***
Sex (fraction male)	2.559 (0.952, 6.879)	0.063		
Age (years)	0.986 (0.950, 1.024)	0.476		
STR	3.733 (1.366, 10.201)	0.010^*^	2.567 (0.746, 8.834)	0.135
Post-operative adjuvant RT	0.453 (0.165, 1.242)	0.124		
Parasagittal and parafalcine	2.110 (0.785, 5.671)	0.139		
Peritumoral edema	0.763 (0.287, 2.024)	0.587		
Calcification	0.350 (0.096, 1.278)	0.112		
Heterogeneous enhancement	0.642 (0.152, 2.721)	0.548		
Cystic change or necrosis	0.888 (0.235, 3.354)	0.861		
Dural tail sign	0.931 (0.347, 2.499)	0.887		
Adjacent bone invasion	10.597 (3.224, 34.831)	< 0.001^*^	7.314 (1.830, 29.239)	0.005^*^
Reactive hyperostosis	1.085 (0.358, 3.291)	0.886		
Multiplicity	3.900 (0.849, 17.922)	0.080		
Maximal diameter (cm)	1.228 (0.947, 1.591)	0.121		
Tumor volume (cm^3^)	1.005 (0.997, 1.014)	0.227		
ADC < 0.825 × 10^−3^ mm^2^/s (cutoff value)	5.752 (1.895, 17.458)	0.002^*^	4.667 (1.335, 16.319)	0.016^*^
SVM score >0.224 (cutoff value)	14.400 (1.855, 111.760)	0.011^*^	8.129 (0.978, 67.569)	0.048^*^

* *Statistical difference (p < 0.05)*.

### Radiomics Approach for the Prediction of P/R

The most significant four imagining features selected by the SVM model for the prediction of P/R were T1 GLCM cluster shade, T1 GLSZM gray-level non-uniformity, T2 GLCM cluster prominence, and T2 GLCM cluster shade. The reproducibility of ROI-based radiomics feature was good, and the ICCs of the four imaging features were 0.92, 0.78, 0.82, and 0.94, respectively.

**Figure 4:**
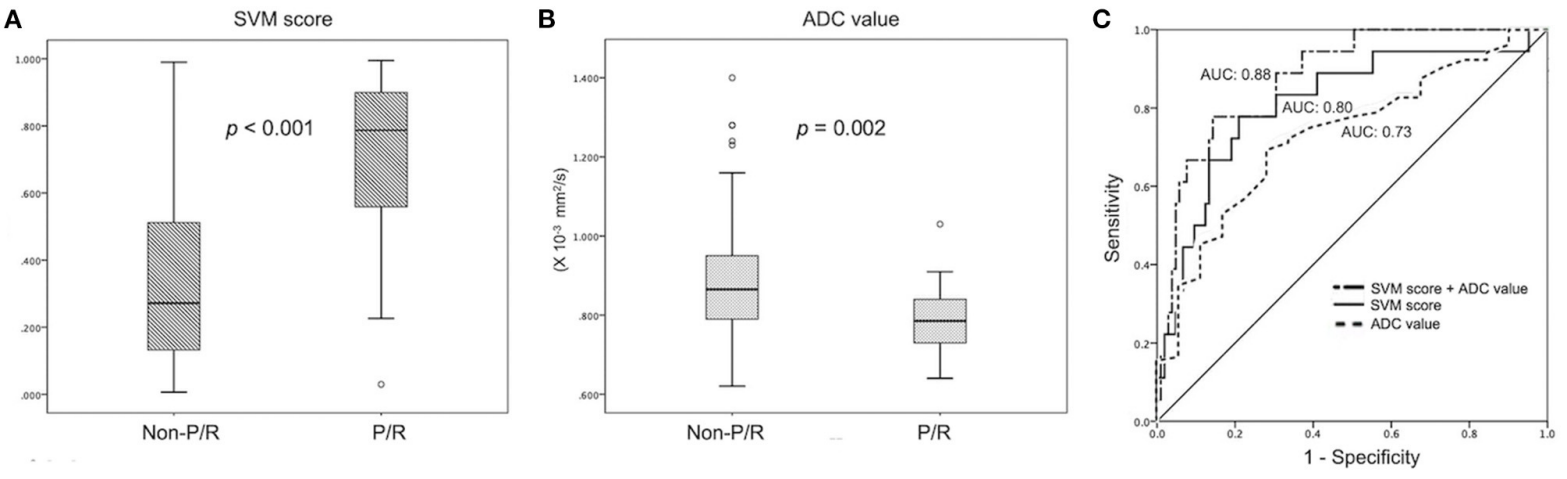
Statistically significant differences (*p* < 0.05) (Mann–Whitney *U* test) are observed in the box plot of **(A)** SVM score and **(B)** ADC value to differentiate between patients with and without P/R. **(C)** Receiver operating characteristic (ROC) curves of SVM score and ADC value for the prediction of P/R in meningiomas, with optimal cutoff value of 0.224 and AUC of 0.825 × 10^−3^ mm^2^/s, respectively. The AUCs of SVM score, ADC value, and combination of SVM and ADC in the prediction of P/R are 0.80, 0.73, and 0.88, respectively.

**Figure 5:**
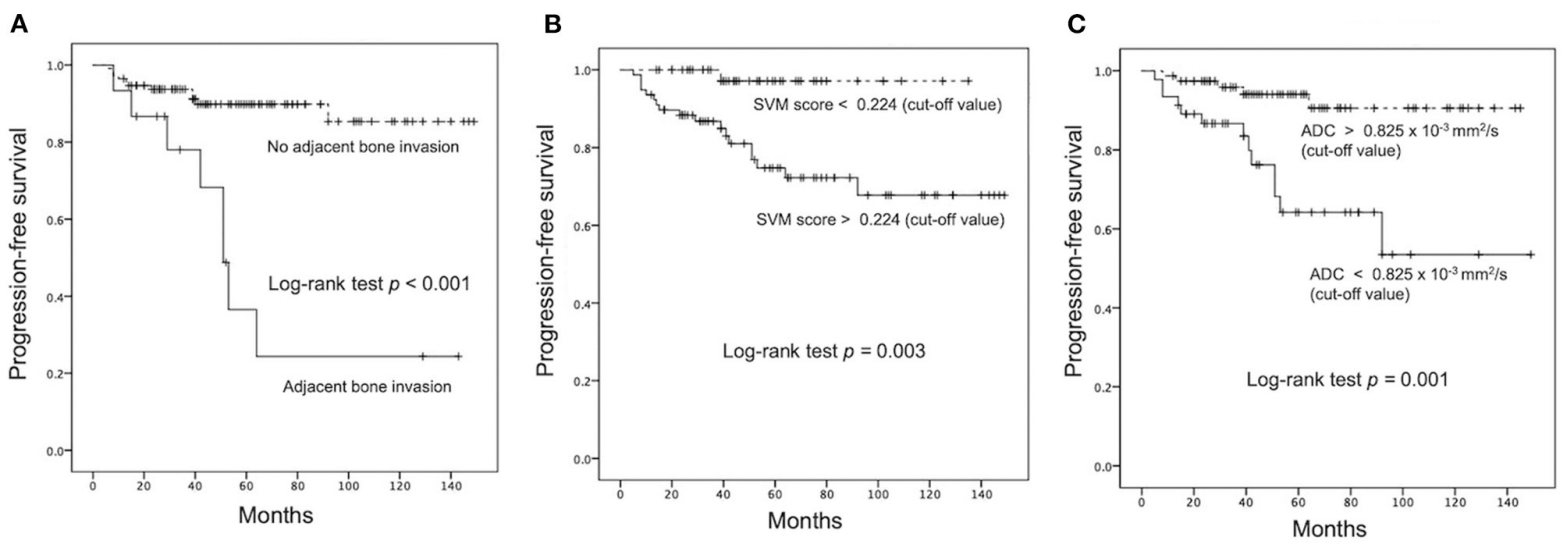
Kaplan–Meier survival curves of **(A)** adjacent bone invasion, **(B)** SVM score, and **(C)** ADC value for the prediction of P/R in meningiomas. All three parameters showed significant difference (*p* < 0.05) (log-rank test) in overall trend of progression-free survival.

For the prediction of P/R, AUCs of 0.80 and 0.73 with optimal cutoff values of 0.224 and 0.825 × 10^−3^ mm^2^/s were obtained in SVM score and ADC value, respectively (Figure 4). Furthermore, improved performance in predictive model was observed after combining SVM score and ADC value, with AUC of 0.88 (Figure 4). When tumor progression trends were compared, patients with adjacent bone invasion, high SVM score (more than the cutoff value of 0.224), and low ADC value (lower than the cutoff value of 0.825 × 10^−3^ mm^2^/s) were found to exhibit shorter PSF (*p* < 0.05) (Figure 5).

## Discussion

In this study, an SVM-based radiomics model was built for the prediction of P/R in meningiomas. A total of 214 first-order and textural features were extracted from CE T1WI and T2WI, and the four most significant features were selected by the SVM algorithm to calculate the personalized SVM score. In multivariate Cox hazards and Kaplan–Meier survival analyses, adjacent bone invasion, low ADC value, and high SVM score were high-risk factors of P/R in meningiomas. For the prediction of P/R in meningiomas, the SVM score-based predictive model is superior to the model based on the ADC value measured manually.

Although 90% of meningiomas are WHO grade I benign tumors, about 21% of these tumors may recur in 5 years after surgical resection (3, 4). Radiomics plays significant roles in the analysis of meningioma characteristics both quantitatively and objectively. Zhu et al. (12) and Chen et al. (34) performed radiomics-based machine learning for pre-operative grading in meningiomas, with AUC of 0.81 and accuracy of 75.6%. Park et al. (8) used the radiomics feature-based machine learning classifiers on conventional and diffusion tensor imaging to predict the grade and histological subtype in meningiomas, with accuracy of 89.7% and AUC of 0.86. Morin et al. (16) integrated radiologic and radiomic features to predict meningioma grade, local failure, and overall survival with AUCs of 0.75 to 0.78. The clinical application of SVM or radiomics score is a new concept. A personalized SVM score could be calculated based on selected radiomic features (35–37). Xu et al. (35) used SVM score to predict pre-operative lymph node metastasis in intrahepatic cholangiocarcinoma, with AUC of 0.87. Fan et al. (38) used SVM-based radiomics score to predict radiotherapeutic response in acromegaly, with AUC of 0.96. Liu et al. (36) reported excellent performance in SVM score to predict treatment response in advanced rectal cancer, with AUC of 0.98. Zhang et al. (10) first applied radiomics to evaluate recurrence in skull base meningiomas, with accuracy of 90%. These studies suggest that SVM score based on radiomics might be a useful tool in predicting recurrences in meningiomas, but rare reports regarding this concept have been published.

Recently, research of computer-extracted radiomic imaging features has become a new field in medical imaging. However, the reproducibility and robustness of the selected imaging features need to be extensively studied before their applications in clinical practice. Factors influencing the robustness of the radiomics approach are modality dependent. However, only few studies have investigated the robustness in MR radiomics (39–42). How different imaging sequences and parameters will affect the reproducibility of radiomic features is still unclear. A recent phantom study showed that obvious differences exist among different MRI sequences in the number of robust and reproducible features (43). However, more than 30% (15 of 45) of the features still showed excellent robustness across all different MR sequences and demonstrate excellent reproducibility. It was supposed that these 15 features can be applied reliably for the design of radiomic models in clinical studies. Among these features, the shape-related feature was noted to be the most robust and reproducible. Only robust and reproducible T1W and T2W radiomic features were suggested to build a radiomics-based model (43). However, it was also true that the effect of operator-dependent bias can be reduced in radiomic features through fully automatic image segmentation as in our study (43).

Lower ADC values have been reported to be associated with a higher rate of recurrence in meningiomas (7). However, subjective ROI placement with various methods for ADC measurement may result in varying results (44). Susceptibility artifact caused by intratumoral calcifications, necrosis, and cystic changes within meningiomas may also interfere with obtaining optimal ADC values (45). The extent of surgical resection is the most important determining factor in the rate of recurrence in meningiomas (46). Nanda et al. (47) reported that the overall recurrence rates of WHO grade I meningiomas in Simpson resection grades I, II, III, and IV are 5, 22, 31, and 35%, respectively. Recurrence rates of 9.7% in the GTR group and 28.6% in the STR group are observed in our study. Although post-operative adjuvant RT improves tumor control status in high-grade meningiomas (48), no standard protocol could be reliably adopted regarding adjuvant RT for benign meningiomas, and clinical practices among different institutions are varied (49). Whether post-operative adjuvant RT will be beneficial for benign meningiomas is still unclear because it increases the risk of complications such as symptomatic peritumoral edema, cranial nerve deficits, and other neurologic deficits (50). Pre-operative radiomics-based prediction for P/R, thus, offers additional information for determining the treatment strategies in meningiomas. For patients with high risks of P/R, aggressive tumor resection in primary surgery combined with post-operative adjuvant RT should be considered. In contrast, the aim of surgery would be the relief of clinical symptoms for other patients to avoid post-operative neurological deficits. Although adjuvant RT may affect the independent predictive value for P/R in our study, no statistically significant difference was observed between the P/R and non-P/R groups.

This study still had several limitations. Selection bias existed due to its retrospective nature. All images are acquired from a single institution, and there is a lack of external validation. Future testing with multi-institutional data and varying imaging protocols is necessary to determine whether the trained predictive classifier is generalizable. The extent of tumor resection and adjuvant RT may affect the independent predictive performance in radiomics analysis although this limitation always exists in studies focusing on this topic due to variations in treatment protocol (6, 7, 10, 32). Because the sample size of P/R is relatively small, only a few imaging features can be selected to build the classification model in order to avoid overfitting. When more cases become available, other machine learning algorithms such as the fully automated convolutional neural network could be implemented. Finally, there is a lack of complete histopathologic findings such as Ki-67 (MIB-1), nuclear atypia, and genomic signature for correlation in this retrospective study.

## Conclusions

Our preliminary study revealed that SVM score based on pre-operative MR radiomic features was a useful tool for the prediction of P/R in meningiomas. Although this was a single institution study, the imaging features extracted based on automatic segmentation and imaging registration were quantitative and objective. Pre-operative MRI radiomics and SVM score, thus, may have the potential to offer valuable information for the planning of treatments in meningiomas, such as the extent of tumor resection, implementation of post-operative adjuvant RT, and the time interval of imaging follow-up. Nevertheless, this method still needs to be validated in a larger-scale study in the future.

## Data Availability Statement

The raw data supporting the conclusions of this article will be made available by the authors, without undue reservation.

## Ethics Statement

The studies involving human participants were reviewed and approved by Institutional Review Board of the Chi Mei Medical Center (IRB no.: 10902-009). Written informed consent for participation was not required for this study in accordance with the national legislation and the institutional requirements.

## Author Contributions

C-CK and J-HC: conceived and designed the experiments. C-CK and YZ: performed the experiments. C-CK, YZ, J-HC, K-TC, T-YC, and S-WL: analyzed the data. T-YC and T-CW: contributed reagents, materials, and analysis tools. C-CK, YZ, and J-HC: wrote the paper. J-HC and M-YS: critically revised the article. All authors contributed to the article and approved the submitted version.

## Conflict of Interest

The authors declare that the research was conducted in the absence of any commercial or financial relationships that could be construed as a potential conflict of interest.
