# Supplementary material for: Pre-operative MRI Radiomics for the Prediction of Progression and Recurrence in Meningiomas
Source: Front Neurol. 2021 May 14;12:636235. doi: 10.3389/fneur.2021.636235 (PMC8160291; doi:10.3389/fneur.2021.636235)
Supplement: Supplementary file 1 [file Table_1.DOCX]

**Radiation therapy techniques**

Adjuvant radiotherapy included stereotactic radiosurgery (SRS) and conventional external beam radiation therapy (EBRT). Conventional EBRT was utilized by intensity modulated radiation therapy (IMRT) and volumetric modulated arc therapy (VMAT) techniques.

1. **Stereotactic radiosurgery (SRS)**

SRS was performed by CyberKnife SRS system (Accuray, Inc., Sunnyvale, CA, USA), using a 6 MV linear accelerator mounted on a fully articulated robotic arm.

1. **Conventional external beam radiation therapy (EBRT)**

IMRT was performed by Elekta Precise (Elekta, Stockholm, Sweden). Treatment plans were generated by the Pinnacle treatment planning system (Philips Medical Systems, Highland Heights, OH). IMRT was delivered with 6 MV X-ray beams modulated coming from proper coplanar angles. VMAT was performed by RapidArc (Varian Medical Systems, Palo, CA), which is one of such VMAT techniques that delivers modulated radiation beams with simultaneous adjustment of multileaf collimator field aperture and gantry rotation speed. Treatment plans were generated using the Eclipse treatment planning system (Varian Medical Systems).
